# Mode I Fracture Toughness of Polyamide and Alumide Samples obtained by Selective Laser Sintering Additive Process

**DOI:** 10.3390/polym12030640

**Published:** 2020-03-11

**Authors:** Dan Ioan Stoia, Liviu Marsavina, Emanoil Linul

**Affiliations:** Department of Mechanics and Strength of Materials, Politehnica University of Timisoara, 1 Mihai Viteazu Avenue, 300 222 Timisoara, Romanialiviu.marsavina@upt.ro (L.M.)

**Keywords:** Three Point Bending test, mode I fracture toughness, selective laser sintering, polyamide and Alumide, geometrical errors, microstructure.

## Abstract

Selective Laser Sintering is a flexible additive manufacturing technology that can be used for the fabrication of high-resolution parts. Alongside the shape and dimension of the parts, the mechanical properties are essential for the majority of applications. Therefore, this paper investigates dimensional accuracy and mode I fracture toughness (K_IC_) of Single Edge Notch Bending samples under a Three Point Bending fixture, according to the ASTM D5045-14 standard. The work focuses on the influence of two major aspects of additive manufacturing: material type (Polyamide PA2200 and Alumide) and part orientation in the building environment (orientations of 0°, 45° and 90° are considered). The rest of the controllable parameters remains constant for all samples. The results reveal a direct link between the sample densities and the dimensional accuracy with orientation. The dimensional accuracy of the samples is also material dependent. For both materials, the angular orientation leads to significant anisotropic behavior in terms of K_IC_. Moreover, the type of material fundamentally influences the K_IC_ values and the fracture mode. The obtained results can be used in the development of additive manufactured parts in order to obtain predictable dimensional tolerances and fracture properties.

## 1. Introduction

The geometrical flexibility of the parts produced by Selective Laser Sintering (SLS) represents the top characteristic of this technology [1,2,3]. Commonly, SLS technology uses the energy of a laser beam in addition to an electrical heating source in order to generate a high enough temperature for particle bonding. Due to the large number of process variables that have to be controlled during the process, some variations of shape, size and mechanical properties of the finite product may occur not only in SLS but also in all additive manufacturing (AM) technologies [4,5,6]. The SLS process variables include chamber temperature, laser energy density, layer thickness, beam offset, shrinkage at cooling, part position and orientation, material type and powder degradation [7]. The deflection from the nominal size and mechanical properties of parts represents a challenging aspect of AM [8,9,10].

Some authors have focused their work on the geometrical accuracy of samples obtained at different orientations and/or energy density by measuring the size and shape and inspecting the surface of the parts by stereomicroscopy [11,12,13] without taking into account the mechanical aspects that are directly influenced by the process parameters. The mechanical characterization of SLS polymer parts was conducted through tensile tests by some authors [14], while in other studies flexural properties were determined according to the manufacturing parameters [15,16]. However, the process parameters greatly influence the mechanical properties of the samples. Efforts in assessing the failure predictions of PA12 samples obtained by SLS have been conducted. The work was based on implementing the failure criterion for AM parts in tensile, compression and shear [17].

To the best of the authors’ knowledge, limited studies cover the fracture behavior of cracked or notched samples manufactured by SLS [18,19,20]. This is a challenging topic since the presence and growing of cracks will interact with the manufacturing layers, sinterization directions and powder particle scattering. Linul et al. [18] used symmetric and asymmetric four-point bending tests to determine the mode I and mode II fracture toughness of laser sintered polyamide. The authors observed that the K_IC_ values were higher than K_IIC_ ones, while the density of the 3D printed samples was highly dependent on the process energy. Fracture mechanics of laser sintered cracked polyamide as a new method to induce cracks was studied by Brugo et al. [19] on six different configurations of Mode I Compact Tension samples. Their results showed that the samples with better mechanical performance were those in which all the layers contained a portion of the crack. On the other hand, Crespo et al. [20] performed experiments at different loading rates to measure the failure loads of different laser sintered notched samples. The results have been used by the authors to apply the TCD to the failure of PA12 notched samples prepared by AM techniques.

The investigation reported here focuses on determining the dimensional accuracy and mode I fracture toughness values of Single Edge Notch Bending Polyamide PA2200 and Alumide samples through a Three Point Bending fixture, designed according to ASTM D5045–14 [21]. Finding out the fracture properties is a step forward, beside tensile and bending properties, in the characterization of AM products. This is of high industrial interest since AM is considered a key technology for the fourth industrial revolution [22]. The mechanical properties of polymers are particularly important because they are generally used as net-shaped parts, unlike metallic components. The fracture properties were underlined in close relation to part orientation inside the building envelope.

## 2. Materials and Methods 

### 2.1. Materials and Specimen Manufacturing

The Polyamide PA2200 powder is a commercially available material produced by Electro Optical Systems (EOS GmbH, Krailling, Germany). This is a multipurpose material that has adequate mechanical (high strength and stiffness) and chemical properties [23]. Long term stability and high detail resolution are also important characteristics of PA2200, making it suitable for a wide range of technical applications [23]. In addition, its biocompatible property makes it usable in custom-made disposable guidance elements, such as those used in the surgical field [24]. Alumide, on the other hand, is a homogeneous mixture of fine polyamide PA12 and fine aluminum particles developed by the same producer. The sintered Alumide possesses a porous structure comparable to polyamide but has a higher stiffness, better thermal conductivity and good density-stiffness ratio [24]. More details regarding the properties and advantages of the PA2200 and Alumide materials are presented in [2].

**Figure 1:**
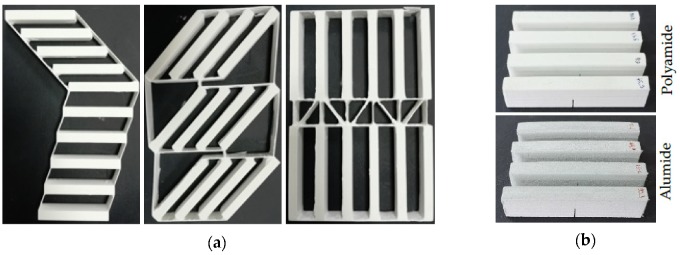
Samples after post-processing oriented at 0°, 45° and 90°, respectively (**a**) and notched polyamide and Alumide samples (**b**).

The additive manufacturing process was conducted on an EOS Formiga P100 (EOS GmbH Electro Optical Systems), on separate stages for each material. The geometry of the specimen was constructed in SolidWorks 2013 according to the specifications of the ASTM D5045–14 standard [21]. The design containing no notches was imported in Materialise Magics 10.0 software [25], where the 3D model was positioned and angularly oriented. The positioning was done using 10 mm clearance between each edge of the parts, and between each part and the machine’s limit. The orientations were only in the XY plane, at values of 0°, 45° and 90° in respect to transversal axis of the machine (X). Ten samples of each material and orientation were manufactured and post-processed by air blasting. In Figure 1a the sintered parts according to their orientations can be observed while Figure 1b depicts the final aspect of both the Alumide and polyamide samples. There is also a visible rib network that was designed to connect the standard part in order to prevent the curling and twisting phenomena as much as possible.

The additive manufacturing of all 60 samples was done using the same process parameters (except the orientation angle) in order to obtain consistent and comparable results. The building chamber and removal chambers were set to 170 °C and 159 °C, respectively. The energy density value was 0.067 J/mm^2^, obtained by setting the power to 25 W, the laser beam velocity to 1500 mm/s and the scan spacing to 0.25 mm. These parameters were chosen based on previous studies and the experience of the authors [2,18,26,27]. A scaling factor of 2.3% was applied for all directions of the sample in order to compensate the shrinkage effect at cooling.

### 2.2. Methods

**Figure 2:**
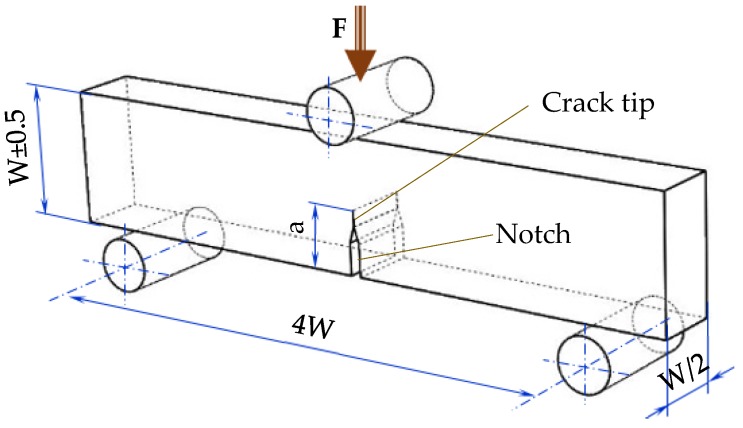
Geometrical parameters of the Single Edge Notch Bend (SENB) sample according to [21].

Prior to crack machining and mechanical testing all samples were measured and weighed. The linear measurements (length L, thickness B and width W) were done using a digital caliper of 0.01 mm precision while the samples masses were measured using a 500 g Kern balance (0.01 g accuracy). In addition, a linear measurement defined as Z deflection was conducted. It characterized the distance from the flat plane supporting the sample to the top of the sample, recorded along the middle line of the sample. The designed samples had a height/width (W) of 20 mm, thickness B = W/2= 10 mm and span length S = 4W = 80 mm (Figure 2). The linear measurements were an indication of curling in the frontal-lateral building planes while the mass and volume of each sample were further used for computing the density.

In order to assess the fracture behavior and to determine the fracture toughness of polyamide and Alumide materials, Single Edge Notch Bend (SENB) samples were adopted in this investigation [21]. The sample notch (8 ± 0.05 mm) was produced using a milling cutting disc with a thickness of 0.6 mm, while the crack (2 ± 0.1 mm) was initiated by using a very sharp and thin razor blade. Therefore, the total length of the crack *a* was about 10 ± 0.15 mm (Figure 2). At least five samples were used for every orientation and each material in the testing phase.

**Figure 3:**
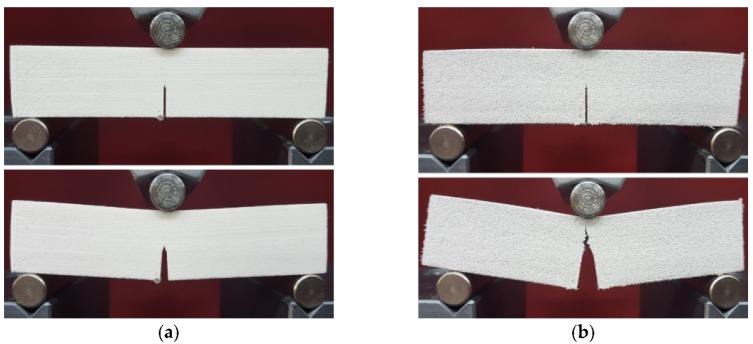
Polyamide (**a**) and Alumide (**b**) SENB samples before and after the quasi-static TPB test.

The SENB samples were tested under a Three Point Bending (TPB) fixture using a Zwick Roell Z005 quasi-static universal testing machine having a maximum load-cell capacity of 5 kN (of 0.1% accuracy). The experimental TPB tests were carried out at room temperature according to the ASTM D5045–14 standard [21]. A constant crosshead speed of 5 mm/min was used, until a fracture occurred. Figure 3 shows the fixing of the SENB samples in the TPB device and the form of the samples before and after the test for the two types of investigated materials, respectively.

The upper loading pin and bottom supports of TPB device were large enough in diameter (10 mm) in order to avoid sample indentation. The load-displacement data were automatically recorded using a sampling frequency of 600 Hz.

Correlations between the orientation input parameter and the output variables (density, fracture toughness and dimensional error) were performed using the conventional computation of Pearson’s correlation coefficient (PCC). The PCC assumes a linear relationship between the input and output parameters and is suitable for small data sets. The PCC’s value will result in the interval [−1, +1], where negative values indicate an inverse relationship between the parameters while a positive value indicates a direct relationship. A value of 1 represents the best correlation while 0 value will indicate no correlation at all [28,29].

The statistical significance of the data obtained for both materials was put in evidence by a probability test (*P*-value). The null hypothesis H0 was defined as no significant difference between the parameter (ρ, K_Ic_, Err.L, Err.B, Err.W,) values of both materials. The alternative hypothesis Ha was defined as the parameter values of the two materials being significantly different. The *P*-values greater than 0.05 (95% confidence) would reject H0 and accept the alternative hypothesis Ha [30,31].

## 3. Results and Discussion

### 3.1. Accuracy Assessment

The length, thickness and width of the specimens were investigated from a form dimensional accuracy perspective. The nominal dimensions of the sample were customized by the scaling factors used for the manufacturing process of 2.3% on each direction. Design dimensions and actual measurements of the sample size were used to calculate the relative error of length, width, thickness and deflection in the Z axis. Each measurement was conducted three times and the mean value for dimension/material at every orientation was used for graphical representation.

**Figure 4:**
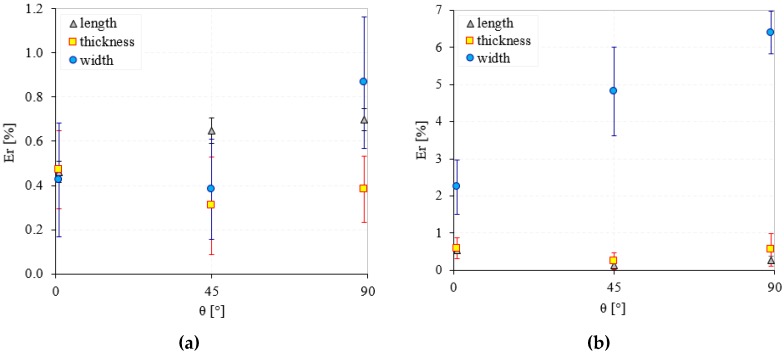
Relative dimensional errors of polyamide (**a**) and Alumide (**b**) samples.

In Figure 4 the relative dimensional errors of the Alumide and polyamide samples are presented as percentages of the design sizes in relation to the angular orientations. Comparable errors were determined in the interval 0.2% to 1.2% for the X-Y manufacturing direction (length and thickness) of both materials. These errors translated in dimensions of 1 to 3 tenths of millimeters, which for an additive manufactured part are acceptable [32,33]. The near error interval was generated by the process symmetry in both sinterization directions and temperature distribution.

By inspecting the dimensional changes of both individual materials on X and Y directions (length and thickness) no trend could be identified in relation to orientation. However, both materials experienced acceptable size errors (below 1%) for the additive manufacturing process [32,33]. The exception was the width error of Alumide that experienced values as large as 6.5% (Figure 4a,b). These high errors for all three orientations revealed an unstable geometry in the z direction. During the sinterization phase, the large expansion of aluminum particles replaced a significant local volume of polyamide powder such that the particle bonding occurred differently than in pure polyamide powder. The aluminum did not participate in the bonding but influenced the bonding formation of polymer particles, conducting finally to changes in structure, dimensions and properties. Furthermore, the uneven distribution of the aluminum particles in the polymer powder generated different local bonding conditions that led to different samples geometries. The phenomenon occurred particularly on the vertical direction because of the free surface of the top layer.

**Figure 5:**
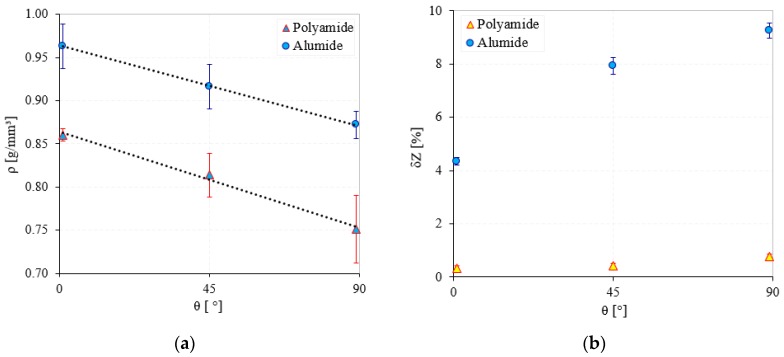
Density (**a**) and Z deflection (**b**) of polyamide and Alumide samples.

In Figure 5 the density of both sintered materials at every orientation angle and the sample deflection in Z direction (width dimension) are presented. The density of the Alumide was higher than the PA due to the aluminum particles that possessed a density 2.2 to 2.3 times higher than PA. 

Regarding the orientation effect, both materials exhibited the same tendency of decreasing the density of the sintered part with the increasing of the orientation angle. This may have been caused by the powder spreading direction in relation to the sample’s directions. A 0° orientation meant that the powder was longitudinally deposited along the length of the samples, while a 90° orientation meant that the powder dispenser blade of the machine was depositing along the thickness of the samples. It appeared that when the dispenser blade spent more time over the object, the powder was better distributed and settled down on the previous layer. The proper powder spreading and slight compaction produced by the dispenser on one hand and the sinterization energy on the other hand led to a higher density of the structure. The deflection error in the Z direction is presented in Figure 5b, showing large values for Alumide (9.1%). The deflection phenomena occurred predominantly during the cooling phase of the process. The larger thermal expansion of the aluminum particles compared with the PA generated larger sample sizes during the sinterization. During the cooling time, the samples shrunk especially in the longitudinal direction. Because the temperature gradient on the top of the samples was different from that on the bottom, the samples bent in Z direction. The differences in the cooling rates on the top and bottom of the samples were generated by the different powder volume (non-sintered) that surrounded the objects and the machine’s architecture.

### 3.2. Fracture Toughness Assessment and Parameter Correlations

**Figure 6:**
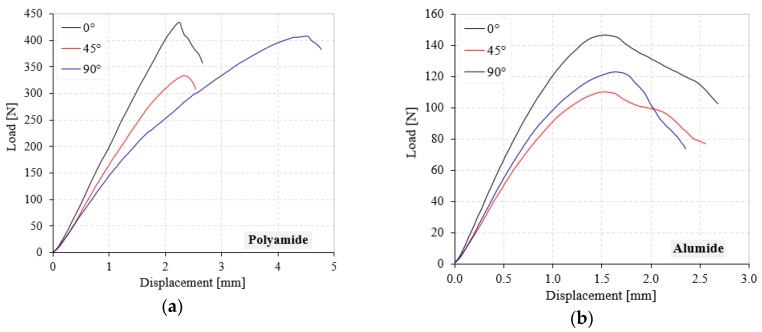
Load-displacement curves of polyamide (**a**) and Alumide (**b**) notched samples.

Following the quasi-static TPB tests, the load (F)-displacement (Δ) curves were obtained and processed. In Figure 6, the F-Δ curves of notched samples are presented for both investigated materials (polyamide and Alumide) and sample orientation angle (0°, 45° and 90°). The F-Δ curves highlighted a linear-elastic behavior that ended with a maximum load, followed by the final fracture of the sample [21]. Beyond the maximum load, the samples either broke sharply or showed a progressive decrease in load carrying capacity, followed by a final fracture. It was seen that the polyamide samples had higher maximum loads than in the case of the Alumide ones. Furthermore, higher loads were required to fracture the samples obtained at 0° orientation, independent of material type. In addition, when we observed the displacement to break, the Alumide had a much more brittle behavior than the polyamide samples. The higher maximum loads and displacements of polyamide compared with Alumide came from the internal structure of these. The pure polyamide material established better bonds between particles, which in the case of Alumide were restricted by aluminum particles. On the other hand, the higher loads recorded for 0° orientation in both materials had to do with the higher densities recorded for this orientation. An explanation for why the density behaved in this way is presented next to the dimensional results.

By using the dimensions of the samples, the mode I fracture toughness (K_IC_) values were determined according to the ASTM D5045–99 standard [21], applying Equation (1).

(1) $$K_{IC}=\frac{F_{crt}}{BW^{0.5}}f\left(\frac{a}{W}\right)\;\left[MPa\cdot m^{0.5}\right]$$

where *F_crt_* is the critical load in [N], determined in accordance with [21], *B* and *W* are geometrical parameters of the samples (thickness and width) in [mm], *a* is the initial crack length of the sample in [mm], while *f(a/W)* is a geometrical function expressed by Equation (2) in terms of *a/W* ratio [21]:

(2) $$f\left(\frac{a}{W}\right)=6\sqrt{\frac{a}{W}}\frac{1.99-\left(\frac{a}{W}\right)\left(1-\frac{a}{W}\right)\left[2.15-3.93\left(\frac{a}{W}\right)+2.7{\left(\frac{a}{W}\right)}^{2}\right]}{\left(1+2\frac{a}{W}\right){\left(1-\frac{a}{W}\right)}^{1.5}}.$$

Table 1 presents the computed mode I fracture toughness values for the two materials (PA2200 and Alumide) for every orientation angle (θ).

**Figure 7:**
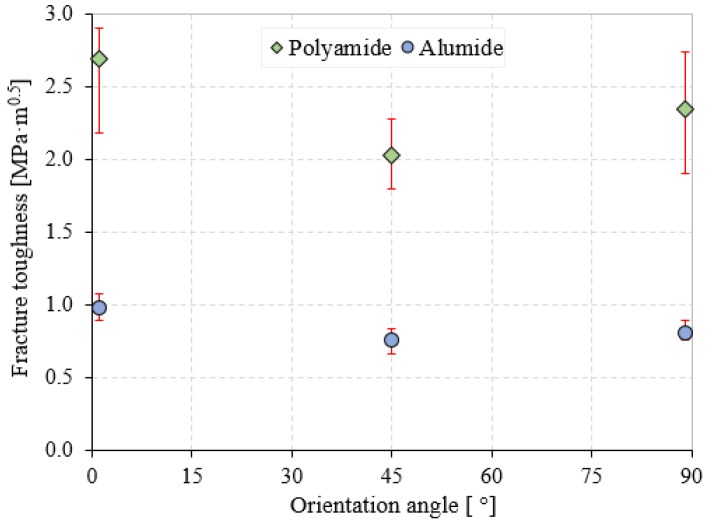
Fracture toughness variation according to the sample orientation angle and material type.

It was obvious that the investigated material had a significant influence on mode I fracture toughness values. Values up to about 63.5% higher in the case of polyamide than in the case of Alumide samples for 0° orientation were obtained, while for the 90° orientation this difference increased up to 65.6%. The smallest difference, of about 62.5%, was observed in the case of a sample orientation angle of 45° (Figure 7). The considerable higher fracture toughness values of pure polyamide versus Alumide were directly connected with the bonding formation between the powder particles in the two materials. Weaker bonding in Alumide was caused by the presence of aluminum particles, which restricted the sinterization bridges formation.

Major differences in K_IC_ values were observed even between the loading directions of the same material. The 0° orientation fracture toughness values were found to be higher than the 90° orientation, with about 12.8% for polyamide and 17.9% for Alumide, respectively. This K_IC_ percentage difference increased by up to 24.4% for polyamide and 22.3% for Alumide between 0° and 45° orientations. Thus, the two materials had approximately the same K_IC_ increases/decreases between different θ. An important aspect to note, which can be seen in Figure 7, is that the polyamide K_IC_ values had much larger error bars compared with the Alumide ones for all sample orientation angles. One of the reasons for these large errors could be related to poor process stability in the case of pure PA compared with Alumide, in which the better thermal transfer conducted a more stable process. Therefore, the investigated materials highlighted a significant anisotropic behavior in terms of mode I fracture toughness, being directly related to the different powder spreading and heat transfer.

**Figure 8:**
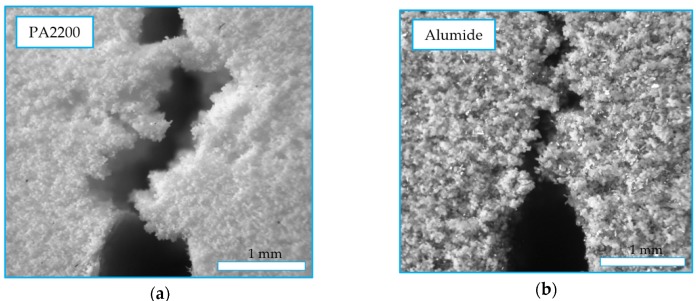
Crack propagation in polyamide (**a**) and Alumide (**b**) samples

Figure 8 presents the crack paths for polyamide and Alumide samples obtained after the TPB fracture test for an orientation angle of 0°. The images were acquired by a Kruss stereo microscope using a magnification of 40X. The crack propagation in both cases followed a relative vertical line, which sustained the mode I fracture.

**Figure 9:**
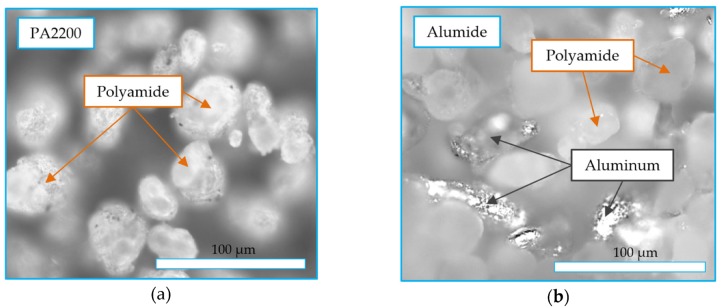
Structure of polyamide (**a**) and Alumide (**b**) samples.

Structure images obtained by optical microscopy of 200X magnification are presented in Figure 9. These revealed the particle bonding of the PA2200 sample (Figure 9a) in the form of small bridges. The identification of the individual particles in the image revealed the sinterization aspect of the process, where coalescence occurred only partially. The image of the Alumide structure revealed some aluminum particles stuck inside the bonded polyamide particles (Figure 9b). The aluminum was not participating to the bond because of its different melting temperature.

In order to establish a relationship between the sample’s orientation angle and the outcome parameters (density, length, thickness and width errors and fracture toughness), Pearson’s correlation was applied to the data. In Table 2 the correlation values are presented, having the following range where -1 is best negative correlation, 0 represents no correlation and +1 is best positive correlation. A very good negative correlation was observed between the sample orientation and the density of the parts, independent of the material type. This was an important finding because the density is a direct consequence of the structure and subsequently to all mechanical properties. Table 2 presents a direct correlation between the fracture toughness and the density, and also good correlations (positive or negative) between the density and the dimensional error.

A low correlation value between *ρ* and K_IC_ in both materials underlined the fact that fracture toughness is not exclusively dependent on the material structure but on the notch manufacturing and accuracy as well. Poor correlation results of Alumide Err B with rho and theta underlined that the effect of temperature differences on the top and bottom of the samples during the cooling phase and the compaction produced by the dispenser influenced the accuracy of this dimension in a much more significant way than rho and theta.

By statistical evaluation of the data, significant differences (*P*-value = 0.003 for densities; *P*-value = 0.007 for fracture toughness) were obtained when comparing the two materials. When the probability test was applied to the dimensional errors, no significant differences were obtained for the investigated materials (*P*-value = 0.275 for length errors; *P*-value = 0.411 for thickness error; *P*-value = 0.07 for width errors). The probability test showed from a statistical perspective that the data we obtained for the two materials were significantly different only for structure and fracture properties, and had no signification when we compared the dimensional error.

## 4. Conclusions

This paper investigates the effect of material type (Polyamide PA2200 and Alumide) and sample orientation angle (0°, 45° and 90°) on mode I fracture toughness (K_IC_) and geometrical accuracy in Selective Laser Sintering.

Size measurements and microstructures were conducted on the samples in order to determine the density and dimensional errors. Three Point Bending tests of Single Edge Notch Bending samples were accomplished for computing the fracture toughness based on force displacement data. In order to give objective interpretations of the recorded data, the probability test (*P*-value) and Pearson’s correlation coefficients were determined.

The density of the samples was greatly influenced by the orientation angle of the samples in both materials, and the best density value was recorded for 0° of orientation. The structural differences between PA2200 and Alumide highly affected the fracture toughness, with the Alumide possessing K_IC_ values of about 62%–65% lower (orientation dependent) than PA2200.

The dimensional accuracy was not significantly different between the considered materials. No matter what the manufacturing angle was, the probability values were above the 0.05 limit. Nevertheless, the dimensional accuracy on every direction had a strong dependence on the manufacturing angle.

## Figures and Tables

**Table 1:** Mode I fracture toughness values of polyamide and Alumide samples according to θ.

Material	Orientation Angle θ [°]	Sample Density ρ [g/cm^3^]	Crack Length a [mm]	Maximum Load F_crt_ [MPa]	Displacement at F_crt_ δ [mm]	Fracture Toughness K_IC_ [MPa/m^0.5^]
**Polyamide**	0	0.855	9.27	335	1.6	2.182
		0.864	9.36	434	2.0	2.902
		0.853	9.63	397	2.3	2.745
		0.861	9.67	407	2.5	2.864
		0.852	9.25	419	2.4	2.753
	45	0.797	8.79	336	2.2	2.063
		0.837	9.20	321	2.0	2.104
		0.766	8.89	287	2.1	1.797
		0.852	9.50	333	2.0	2.276
		0.782	9.93	266	1.6	1.924
	90	0.717	9.36	315	1.6	1.907
		0.737	8.90	350	3.3	2.208
		0.764	8.63	409	3.9	2.464
		0.759	9.76	335	1.7	2.405
		0.781	9.03	427	4.3	2.743
**Alumide**	0	0.957	9.52	136	1.5	0.897
		0.979	9.62	147	1.5	0.982
		0.987	9.78	158	1.5	1.079
		0.970	9.48	140	1.6	0.929
		0.982	9.78	148	1.5	1.020
	45	0.854	10.67	99	1.3	0.713
		0.878	10.56	96	1.3	0.713
		0.881	10.24	110	1.6	0.783
		0.886	10.37	118	1.6	0.848
		0.879	10.36	110	1.5	0.755
	90	0.901	9.88	121	1.3	0.834
		0.878	10.30	99	1.2	0.708
		0.939	9.74	127	2.0	0.885
		0.899	10.23	104	1.4	0.739
		0.915	10.02	123	1.6	0.865

**Table 2:** Pearson’s correlation between sample orientation, density, size and fracture toughness.

Material/Parameter	Polyamide		Alumide	
	θ [°]	ρ [g/mm^3^]	θ [°]	ρ [g/mm^3^]
ρ [g/mm^3^]	−0.996	1	−0.999	1
K_IC_ [MPa/m^0.5^]	−0.523	0.448	−0.755	0.764
Err. L [%]	0.946	−0.918	−0.644	0.661
Err. B [%]	−0.530	0.461	−0.098	0.118
Err. W [%]	0.828	−0.869	0.989	−0.992
